# Risk Factors for Postoperative Cerebrospinal Fluid Leak Following Endoscopic Transsphenoidal Surgery for Craniopharyngioma: A Multicenter Cohort Study with a Contemporary Surgeon Practice Survey

**DOI:** 10.21203/rs.3.rs-9285619/v1

**Published:** 2026-04-07

**Authors:** Sandhya R Palit, Yuki Shinya, Maria Peris Celda, Michael Karsy, James J Evans, Michael R Chicoine, Albert H Kim, Bhuvic Patel, Won Kim, Marvin Bergsneider, Donato R Pacione, Paul Gardner, Raj Mukherjee, Connor Liu, Stephanie Cheok, Nathan T Zwagerman, Charles Christenson, Spiros Blackburn, Varun R Kshettry, Robert C Rennert, William T Couldwell, Ramin Morshed, Kyle C Wu, Daniel Prevedello, Garni Barkhoudarian, Juan C. Fernandez-Miranda, Gabriel Zada, Carolina Benjamin, Michael P Catalino, Adam Mamelak, Andre Beer Furlan, Georgios Zenonos, Michelle M Mendoza, Andrew S Little, Jamie J. Gompel

**Affiliations:** Mayo Clinic; Mayo Clinic; Mayo Clinic; University of Michigan Health; Thomas Jefferson University; University of Missouri Health Care; Washington University School of Medicine; Washington University School of Medicine; UCLA Health; UCLA Health; NYU Grossman School of Medicine; NYU Grossman School of Medicine; Johns Hopkins University School of Medicine; Johns Hopkins University School of Medicine; Medical College of Wisconsin; Medical College of Wisconsin; The University of Texas Health Science Center at Houston; The University of Texas Health Science Center at Houston; Cleveland Clinic; University of Utah Health; University of Utah Health; University of California, San Francisco; The Ohio State University; The Ohio State University; Saint John’s Cancer Institute at Providence Saint John’s Health Center, Pacific Neuroscience Institute; Stanford University School of Medicine; Keck School of Medicine of USC; University of Miami Miller School of Medicine; University of Virginia; Cedars-Sinai Medical Center; Moffitt Cancer Center; University of Pittsburgh School of Medicine; Barrow Neurological Institute, St. Joseph’s Hospital and Medical Center; Barrow Neurological Institute, St. Joseph’s Hospital and Medical Center; Mayo Clinic

**Keywords:** Craniopharyngioma, Skull Base Reconstruction, CSF Leak, Surgical Practice, Endoscopic Transsphenoidal Surgery, Surgeon Survey

## Abstract

**Purpose:**

This multicenter, multi-surgeon retrospective study aimed to identify risk factors for postoperative cerebrospinal fluid (CSF) leak following endonasal resection of craniopharyngioma, while evaluating the “perception gap” through a surgeon survey.

**Methods:**

A retrospective review was conducted on 416 patients who underwent endoscopic transsphenoidal surgery (ETS) for craniopharyngioma from 20 institutions. Factors were compared between patients with and without postoperative CSF leak, and between Early (2007-2015) and Late (2016-2025) Epochs. Complementing the clinical data, a survey of 19 neurosurgeons captured expert perspectives on risk stratification and management strategies.

**Results:**

Overall postoperative CSF leak rate was 13.5% (56/416 patients). Univariate analysis identified predominantly cystic tumors (34% vs. 21%, *p* = 0.034)and intraoperative lumbar drain (LD) use (*p* < 0.028) as associated with postoperative CSF leak. BMI (*p* = 0.587), prior surgery (*p* = 0.576), and tumor size (*p* = 0.363) were not significant. In the multivariable analysis, LD use was associated with a higher postoperative CSF leak rate (OR 1.91, 95% CI 1.06–3.46; *p* = 0.030). Between Epochs, nasoseptal flap (NSF) utilization increased from 70.7% to 87.6% (*p* <0.001). NSF was protective (OR 0.28, 95% CI 0.07–0.92; *p* = 0.037) in the Early Epoch; no factors were significant in the Late Epoch. The surgeons’ survey identified prior surgery and intraoperative high flow leaks as primary risks; however, their LD protocols diverged from clinical data.

**Conclusion:**

Despite increased use of NSF, CSF leak persists. The study highlights a significant divergence between expert perception and clinical data regarding lumbar drains.

## INTRODUCTION

Craniopharyngiomas pose a unique surgical challenge due to their intimate relationship with critical neurovascular structures, frequent suprasellar extension, and propensity for cystic degeneration.^[1]^ Endoscopic transsphenoidal surgery (ETS) has emerged as a preferred approach in many cases, offering superior visualization and access to the suprasellar space compared with microscopic or transcranial techniques.^[2–6]^ Nevertheless, postoperative cerebrospinal fluid (CSF) leak remains a critical and frequent complication with reported incidences around 15–20% in contemporary series.^[7–12]^ Hence, identifying patient specific and tumor characteristics that predispose to CSF leak is paramount to effective preoperative risk stratification.

Several preoperative and intraoperative factors have been suggested to influence the risk of postoperative CSF leak, including tumor size, pattern of suprasellar extension, tumor consistency, prior treatment, and the choice of reconstruction strategy.^[7, 8, 10–13]^ The introduction of the vascularized nasoseptal flap and multi-layered skull base reconstruction has markedly improved postoperative outcomes in ETS for craniopharyngioma.^[2, 14–16]^ However, heterogeneity in techniques, variation in surgeon experience, and inconsistent adoption of multilayer reconstruction or adjunctive materials continue to contribute to fluctuating CSF leak rates across institutions.^[7, 13, 17]^ Moreover, the role of lumbar drains (LD) remains controversial, with conflicting evidence regarding their utility and risks, including over-drainage and meningitis.^[18–20]^

A persistent gap in literature concerns the relationship between objective clinical risk factors and surgeon perception.^[17, 21]^ While cohort-based analyses identify statistical predictors of postoperative complications, they do not reflect how surgeons anticipate or manage these risks. Conversely, practice surveys highlight variations in management strategies but lack validation against patient-level outcomes.^[13, 17]^ Addressing this disconnect is essential for establishing more consistent, evidencebased standards for reconstruction and perioperative care.

This study aims to address these clinical and statistical gaps by integrating a multicenter retrospective cohort analysis with a complementary survey of 19 experienced skull base neurosurgeons amongst 20 centers. The primary objective is to identify independent risk factors for postoperative CSF leak following ETS for craniopharyngioma. The secondary objective is to evaluate the divergence between objective risk factors and surgeon-reported management practices, thereby elucidating contemporary practice patterns and guiding future efforts toward standardization.

## METHODS

### Study Design and Patient Cohort

A multicenter retrospective cohort study was conducted across 20 institutions participating in the Registry for Adenomas of the Pituitary and Related Disorders (RAPID) Consortium.^[22, 23]^ Patients who underwent ETS for craniopharyngioma between 2007 and 2025 were retrospectively identified. This study was conducted in accordance with the Declaration of Helsinki and institutional review board approval was obtained at all participating centers. Patients were eligible if they had histopathologically confirmed craniopharyngioma, underwent ETS as the surgical approach, and had a minimum postoperative follow-up of 90 days.

All variables were determined and reviewed by a core steering committee of the RAPID Consortium, ensuring high data quality and consistency across multiple institutions. The details of the consortium and data sharing have been previously reported.^[24]^ Patient demographics, including age, sex, preoperative comorbidities, and prior treatments, were collected. Tumor characteristics were assessed using preoperative magnetic resonance imaging (MRI) data with maximum tumor diameter, tumor consistency (cystic component only, solid, and mixed), tumor location (sellar, suprasellar, intraventricular), relation to pituitary stalk (pre-infundibular, post-infundibular, trans-infundibular), and relation to optic nerve (pre-chiasmatic, post-chiasmatic). Intraoperative variables included the involvement of an otolaryngologist, the use of an LD, and details of reconstruction such as the use of a nasoseptal flap, fat graft, fascia lata, or other layered techniques. To evaluate the impact of evolving surgical standards and the standardization of reconstructive techniques over time, the cohort was stratified into roughly equivalent halves: an Early Epoch (2007–2015) and a Late Epoch (2016–2025). This division allows for a comparison between the early adoption phase and the contemporary period of standardized multi-layered reconstruction.^[25]^

### Data Analysis and Risk Factor Assessment

The primary outcome was a postoperative CSF leak within 90 days of ETS. Secondary outcomes included postoperative meningitis, need for revision surgery, and the occurrence of new endocrine deficit. Statistical analyses were performed using Bluesky Statistics (version 10.3.4). Given the large volume and complexity of the dataset, all analyses were independently reviewed by institutional statisticians for methodological validation. Categorical variables were compared using Chi-square (Fisher’s exact test where appropriate). Continuous variables were evaluated using the Mann–Whitney U test (Wilcoxon rank-sum test), including an assessment of BMI distributions between lumbar drain and non-lumbar drain cohorts to evaluate for confounding by indication. To maintain model parsimony and adhere to the events-per-variable principle, we prioritized variables with known clinical impact on skull base reconstruction for multivariable analyses. Statistical significance was defined as *p* < 0.05 in accordance with the large number of variables assessed.

### Surgeon Survey across RAPID Consortium

To contextualize these findings, a complementary, multi-institutional survey was administered to 19 experienced pituitary and skull base surgeons across RAPID Consortium to capture current practice patterns and management strategies.^[22]^ Surgeons were asked to rank the perceived importance of specific preoperative and intraoperative risk factors and the preference for reconstruction materials. The mean rank for each factor was calculated by averaging the individual rank assigned by each surgeon. The Likert ranking scale ranged from 1 to 5, where a rank of 1 indicated ‘Extremely Importan’ or ‘Always Used,’ and a rank of 5 indicated ‘Not at all Importan’ or ‘Never Used.’ Data on the sequential steps for managing a condfirmed postoperative CSF leak and the preferred interventions for managing increased intracranial pressure were collected. The sequence of interventions was determined by the lowest numerical average rank, signifying the highest clinical priority. These reported practices were summarized descriptively to explore any observed divergence in contemporary practice from the clinical data.

## RESULTS

### Baseline characteristics and Outcomes

A total of 416 patients who underwent ETS for craniopharyngioma met inclusion criteria. The overall rate of postoperative CSF leaks within 90 days was 13.5% (n=56/416). Baseline characteristics stratified by the presence or absence of a postoperative CSF leak are summarized in Table 1. Patients who developed a postoperative CSF leak were more likely to have predominantly cystic tumors than in those without postoperative CSF leak (33.9% vs. 21.1%, *p* = 0.034). Intraoperative use of a lumbar drain (LD) was significantly higher in the CSF leak group compared to the non-leak group (57.1% vs. 38.9%; *p* = 0.028) (Table 1). In a subgroup analysis focusing on patients with prior radiation (n=36), the postoperative CSF leak was 0% (0/12) in those who received an intraoperative lumbar drain compared to 20.8% (5/24) in irradiated patients who did not receive a lumbar drain. This difference did not reach statistical significance (*p* = 0.146, Fisher’s Exact Test).

A multivariable logistic regression analysis utilizing five predictors was performed to identify independent predictors of postoperative CSF leak (Table 2). In the final adjusted model, lumbar drain use was the only variable that reached independent statistical significance (OR 1.91, 95% CI 1.06–3.46; *p* = 0,030) (Table 2).

### Evolution of Reconstructive Practices – Epoch Analysis

The cohort was stratified into an Early Epoch (2007-2015, n=75) and a Late Epoch (2016-2025, n=340) (Table 3). The overall postoperative CSF leak rate was 18.7% in the Early Epoch and 12.4% in the Late Epoch (*p* = 0.147). The utilization of the Nasoseptal flap increased from 70.7% in the Early Epoch to 87.6% in the Late Epoch (*p* < 0.001). Similarly, the use of fascia lata significantly increased from 22.7% to 35.3% (*p* = 0.035). The rate of lumbar drain utilization remained constant across both epochs, at 42.7% and 41.2%, respectively (*p* = 0.813) (Table 3).

In the Early Epoch, both the Nasoseptal flap and lumbar drain were significant predictors of CSF leak on bivariate analysis (Table 4). The use of an NSF was associated with a significantly lower risk of leak (OR 0.22, 95% CI 0.06–0.74; *p* = 0.016), while LD use was associated with over fourfold increased odds of a leak (OR 4.43, 95% CI 1.31–17.72; *p* = 0.021).

In the multivariable analysis for the Early Epoch, only the Nasoseptal Flap remained an independent protective factor, associated with a 72% reduction in the odds of a CSF leak (OR 0.28, 95% CI 0.08–0.97; *p* = 0.045). No factors were significantly associated with CSF leak in the multivariable model for Late Epoch (Table 4).

### Surgeon Perception, Reconstruction, and Management

Survey responses from 19 expert pituitary and skull base neurosurgeons who contributed to this study were summarized in Tables 5. Among preoperative factors, prior surgery (average rank 1.80), patient comorbidities (1.94), tumor location (2.00) and prior radiation therapy (2.05) were identified as risk factors for postoperative CSF leak (Table 5). Intraoperative CSF leak (1.74) was ranked as a high-risk intraoperative contributor; and reconstruction practices demonstrated the Nasoseptal flap as the preferred primary reconstructive technique (average rank 1.16). The decision to use specific reconstruction materials was highly influenced by surgeon preference (12/19 respondents), tied with objective factors such as tumor location, Esposito graded repair approach^[15]^, prior treatment, and tumor size (11/19 respondents each) (Table 5). Regarding management of confirmed postoperative CSF leak, the average reported priority of order of intervention was imaging confirmation (1^st^), endoscopic visualization (2^nd^), antibiotic coverage (3^rd^), a lumbar drain trial (4^th^), and finally, immediate return to surgery (5^th^) (Table 5).

## DISCUSSION

### Key results

This multicenter study leverages one of the largest contemporary cohorts of ETS for craniopharyngioma to analyze the risk factors for postoperative CSF leak, utilizing data from 20 participating institutions within the RAPID consortium. The overall postoperative CSF leak rate was **13.5%**, with a decrease observed from **18.7%** in the Early Epoch to **12.4%** in the Late Epoch. Univariate analysis identified **primarily cystic tumors** as having a higher association with postoperative leaks (**33.9% vs. 21.1%, *p* = 0.034**). While the use of nasoseptal flap was identified as an independent protective factor in the Early Epoch (OR 0.22, *p* = 0.020), it was no longer significant in the Late Epoch as nasoseptal flap reached near universal utilization of 90%. Additionally, the use of **fascia lata** also increased significantly over the study period (**22.7% to 35.3%, *p* = 0.035)**. In the overall multivariable model, **lumbar drain (LD) use** was the only independent predictor of a leak (**OR 1.91, *p* = 0.030**), though its statistical risk association was decreased over time.

### Interpretation

While previous studies have identid higher BMI (36.3 – 39.2 kg/m^2^) as a significant risk factor for postoperative CSF leak due to increased intracranial pressure,^[26, 27]^ our findings align more closely with series where BMI was not a significant predictor.^[11]^ In our cohort, the overall median BMI was nearly identical between patients with and without a leak (**29.8 vs. 28.7; *p* = 0.587**). However, we believe this lack of association reflects a significant **confounding by indication** rather than a lack of physiological impact. Our analysis revealed that patients receiving intraoperative LDs had a higher median BMI compared to those who did not (**30.36 vs. 28.28; *p* = 0.145**), suggesting that clinicians were highly effective at identifying high-risk physiological profiles and selectively employing drainage to mitigate perceived pressure risks. This supports the hypothesis that surgeons utilize a ‘gestalt’ assessment to identify patients with high-risk physiological profiles and proactively manage them with CSF diversion.

Beyond patient-specific factors, tumor morphology plays a critical role in reconstructive success. Our univariate analysis identified **primarily cystic tumors** as having a significantly higher association with postoperative CSF leaks compared to solid or mixed variants (33.9% vs. 21.1%, *p* = 0.034). This finding is relevant given that craniopharyngioma cystid contains high concentrations of cholesterol crystals and inflammatory cytokines, which are known to trigger **aseptic meningitis** and **cerebral vasospasm**.^[28]^ The chemical irritation caused by the intraoperative spillage of this ‘machine oil’ fluid may incite a robust inflammatory response at the skull base, potentially compromising the integrity of the nascent reconstructive seal. Conversely, such inflammation could theoretically promote healing, but our data suggests a clinical association with higher leak rates in predominantly cystic variants.^[29]^

The stratification of the cohort into Early and Late Epochs allowed for a granular assessment of how surgical practice and reconstructive standards shifted over time. The transition of the nasoseptal flap from a potent independent protective factor in the Early Epoch (*p* = 0.045) to a non-significant variable in the Late Epoch (*p* = 0.998) illustrates its evolution from a selective reconstruction material to the foundation of standardized care.^[15, 17]^ This suggests that its widespread adoption may have effectively neutralized the nasoseptal flap as a statistical variable, aligning clinical practice with high expert consensus (rank 1.16) observed in our survey.

Beyond the nasoseptal flap, the utilization of other reconstruction materials in multilayer skull base repair warrants consideration.^[12, 14, 30]^ While the use of fascia lata also increased significantly from 22.7% to 35.3% (*p* = 0.035), neither fascia lata nor fat graft use emerged as independent predictors of postoperative CSF leak in the Late Epoch. This suggests a hierarchical approach to reconstruction; while the nasoseptal flap serves as the foundation of standardized care for high-flow defects, the elective addition of secondary adjunctive materials may be governed more by specific intraoperative findings or individual surgeon preference than by statistical requirement for a success.^[16, 31–33]^

The role of perioperative lumbar drains remains controversial, and our data contributes to this ongoing discussion.^[11, 18–20, 34, 35]^A comparison of the two surgical eras reveals a significant evolution in lumbar drain utilization patterns and their associated risks. In the Early Epoch, the risk of CSF leak persisted despite the concurrent use of lumbar drain as demonstrated by a potent bivariate association (OR 4.43, 95% CI 1.31–17.72; *p* = 0.021), however, by the Late Epoch there was no association (Bivariate OR 1.68, *p* = 0.118; Multivariable OR 1.45, *p* = 0.287). This reduction in the odds ratio suggests a shift in confounding by indication.^[17]^ Surgeons are highly effective at identifying high-risk patients and selectively employing LDs in those cases. This is further supported by the aforementioned finding that patients receiving LDs had a higher median BMI (30.36 vs. 28.28, *p* = 0.145), suggesting that clinicians preferentially utilize LD in patients with physiological profiles perceived to carry higher intracranial pressure and leak risk. This high-level, individualized approach to patient care in the modern epoch suggests that surgeon-directed risk mitigation may have decreased the risk of traditional failure factors such as elevated BMI and complex tumor characteristics.

Furthermore, the clinical context of “intraoperative” placement warrants consideration, as the decision to insert an LD may occur either at the onset of surgery or as a reactive measure at the end of the procedure following the observation of a high-flow defect. This reactive usage in the most challenging cases likely masks any primary protective benefit of the drain. The clinical efficacy of this selective approach is contextualized by two landmark prospective studies. While **Zwagerman et al. (2018)** established through a randomized controlled trial that prophylactic LDs significantly reduce leak rates in high-flow defects, subsequent analysis by **Lavigne et al. (2022)** demonstrated that **selective implementation** based on surgeon intuition yielded even superior outcomes compared to routine protocols.^[34, 36]^ Our survey results reinforce this surgeon preference model; while experts prioritize the LD for high-risk scenarios and confirmed leaks, there is a clear paradigm shift toward selective rather than routine application, underscoring the dominance of individualized risk assessment in contemporary skull base reconstruction.

The reported management sequence for a confirmed leak prioritizes antibiotic coverage and a lumbar drain trial before immediate return to surgery. The prioritization of early antibiotics likely reflects a cautious clinical paradigm around bacterial meningitis aimed at preventing rapid neurological deterioration and escalation into life-threatening sepsis or permanent deficit if not addressed immediately. While the immediate use of antibiotics serves as a critical safety net against rapid decline, our findings suggest that prophylactic antibiotics must be used judiciously. Given that aseptic meningitis (driven by the aforementioned inflammatory cyst fluid) accounts for 50% to 75% of postsurgical febrile cases, a more nuanced approach is required to distinguish chemical irritation from true bacterial.^[29, 37]^ However, this sequence is also conflicting because existing literature strongly links prolonged LD use to an extremely high incidence of infection, with some studies reporting up to a 23-fold increase in the odds of meningitis and suggests this intermediate step may unnecessarily delay definitive surgical repair of the defect.^[17–19, 34, 38, 39]^

By combining a multicenter retrospective analysis with a detailed survey of experienced skull base surgeons, this study identifies not only objective risk factors but also the “perception-data gap,” providing a more holistic view of surgical decision-making than cohort studies alone.

### Limitations

This study is limited by its retrospective design, which may introduce selection and documentation bias. Variability among institutions regarding imaging protocols and reconstruction techniques may influence outcomes. Our dataset lacks granular detail regarding secondary reconstructive materials; specifically, we could not distinguish between intradural (inlay) and extradural (onlay) placement of fascia lata. This distinction is vital, as biomechanical success often depends on the spatial orientation of these layers relative to the defect.

Furthermore, data on intraoperative lumbar drain (LD) use did not distinguish between prophylactic and reactive placement. LD management is also highly heterogeneous regarding drainage volume and duration, variables not captured in this registry. This prevents a definitive assessment of whether the observed risk association is entirely due to confounding by indication, where LDs are selectively used in high-risk cases. Additionally, unmeasured variables, such as Esposito leak grades or specific surgeon maneuvers, remains a potential confounder. Finally, the survey reflects practices of experienced tertiary-care surgeons and may not generalize to all centers.

## CONCLUSION

This study highlights an evolution in craniopharyngioma management, transitioning from selective adjuncts to standardized, high-quality reconstruction. Near-universal adoption of the nasoseptal flap has successfully mitigated anatomical and clinical challenges that historically predisposed patients to postoperative CSF leaks. The divergence between expert consensus and clinical outcomes regarding lumbar drains underscores the impact of confounding by indication. Future efforts must align preoperative risk perception with these modern clinical realities to further optimize outcomes.

## Figures and Tables

**Table 1:** Baseline Comparisons of Patients Characteristics

Variables		CSF Leak (N=56)	No CSF Leak (N=360)	Total (N=416)	*p* - value
**Age (median, SD)**		54 (17.03)	48 (39.32)	48.2 (37.09)	0.163
**BMI (median, SD)**		29.8 (7.6)	28.8 (7.68)	28.8 (7.82)	0.587
**Gender, Females**		31 (55.4%)	151 (41.9%)	182 (43.8%)	0.060
**Prior Surgical Management**		14 (25%)	78 (21.7%)	92 (22.1%)	0.576
**Prior Radiation**		5 (8.9%)	31 (8.6%)	36 (8.7%)	0.937
**Tumor Characteristics on Imaging**					
**Max tumor diameter, cm**		2.4 (1.58)	2.6 (2.46)	2.6 (2.36)	0.363
**Tumor size groups, cm**	**0-2.0**	19 (33.9%)	96 (26.7%)	115 (27.6%)	0.461
	**2.1-3.0**	19 (33.9%)	148 (41.1%)	167 (40.1%)	
	**>3.1**	18 (32.1%)	116 (32.2%)	134 (32.2%)	
**Tumor consistency**	**Primarily Cystic**	19 (33.9%)	76 (21.1%)	95 (22.8%)	**0.034** *
	**Solid and Mixed**	37 (66.1%)	284 (78.9%)	321 (77.2%)	
**Intraoperative and Reconstruction Materials Used**					
**Lumbar Drain Use**		32 (57.1%)	140 (38.9%)	172 (41.3%)	**0.028** *
**NSF used**		42 (75.0%)	310 (86.1%)	352 (84.6%)	0.084
**Fat Graft used**		26 (46.4%)	125 (34.7%)	151 (36.3%)	0.127
**Fascia Lata used**		21 (37.5%)	116 (32.2%)	137 (32.9%)	0.420

Continuous variables (Age, Basal Metabolic Rate) are presented as median values. Categorical variables are presented as n (%). Statistical analysis was performed using Pearson’s Chi-square test (for categorical variables) or the Mann-Whitney U test (for continuous variables). BMI, Body Mass Index; CSF, Cerebrospinal Fluid; cm, centimeter, NSF, nasoseptal Flap;

* *p* value < 0.05 was considered significant.

**Table 2:** Bivariate and Multivariable Logistic Regression Analysis of Risk Factors for Postoperative Cerebrospinal Fluid Leak

Variables	Bivariate		Multivariable	
	*p* - value	OR (95% CI)	*p* - value	OR (95% CI)
**Primarily Cystic Tumor Consistency**	**0.035** *	1.91 (1.02–3.49)	0.151	1.58 (0.83–2.95)
**Nasoseptal Flap Use**	**0.035** *	0.48 (0.25–0.97)	0.143	0.59 (0.30–1.22)
**Lumbar Drain Use**	**0.011** *	2.10 (1.19–3.73)	**0.030** *	1.91 (1.06–3.46)
**Age**	0.660	1.00 (0.99–1.01)	0.612	1.00 (0.99–1.01)
**Gender, male**	0.061	0.58 (0.32–1.02)	0.093	0.60 (0.33–1.08)

The Multivariate model adheres to the Events Per Variable principle (50 events/5 predictors). Age was included as a continuous variable (per year increase). The remaining predictors were entered as binary categorical variables where the reference group represents the absence of the factor, specifically: Tumor Consistency (Primarily Cystic vs. Mixed or Solid), Nasoseptal Flap Use (Used vs. Not Used), Lumbar Drain Use (Used vs. Not Used), and Gender, male (Male vs. Female). OR, Odds Ratio; CI, Confidence Interval;

* *p* value < 0.05 was considered significant.

**Table 3:** Evolution of Reconstructive Technique and CSF Leak Rate in Early (≤ 2015) vs. Late (> 2016) Epochs

Variables		Early 2007-2015 (N=75)	Late 2016-2025 (N=340)	*p* - value
**BMI (median, SD)**		28.27 (9.06)	29.11 (7.47)	0.556
**Age (median, SD)**		45 (78.40)	50 (18.56)	0.158
**Max tumor diameter, cm (median, SD)**		1 (0.490)	1(0.500)	0.158
**Tumor Size Groups, cm**	**0-2.0**	20 (26.7%)	94 (27.6%)	0.973
	**2.1-3.0**	30 (40%)	137 (40.3%)	
	**>3.1**	25 (33.3%)	109 (32.1%)	
**Tumor Consistency**	**Primarily Cystic**	23 (30.7%)	72 (21.2%)	0.077
	**Solid and Mixed**	52 (69.3%)	268 (78.8%)	
**Perioperative Lumbar Drain**		32 (42.7%)	140 (41.2%)	0.813
**NSF**		53 (70.7%)	298 (87.6%)	**<0.001** *
**Fat Graft**		32 (42.7%)	118 (34.7%)	0.194
**Fascia Lata**		17 (22.7%)	120 (35.3%)	**0.035** *
**Postoperative CSF Leak**		14 (18.7%)	42 (12.4%)	0.147

Categorical variables are presented as n (%). Statistical analysis was performed using Pearson’s Chi-square test (for categorical variables) or the Mann-Whitney U test (for continuous variables). BMI, Body Mass Index; CSF, Cerebrospinal Fluid; NSF, nasoseptal Flap; cm, centimeter;

* *p* value < 0.05 was considered significant.

**Table 4:** Multivariable Logistic Regression Analysis of Risk Factors for Postoperative CSF Leak in the Early Epoch (2007-2015) and the Late Epoch (2016-2025).

Variables	Bivariate		Multivariable	
	*p* - value	OR (95% CI)	*p* - value	OR (95% CI)
**Early Epoch (2007-2015)**				
**Nasoseptal Flap Use**	**0.016** *	0.22 (0.06–0.74)	**0.045** *	0.28 (0.08–0.97)
**Lumbar Drain Use**	**0.021** *	4.43 (1.31–17.72)	0.055	3.63 (1.02–14.98)
**Late Epoch (2016-2025)**				
**Nasoseptal Flap Use**	0.685	0.82 (0.35–2.29)	0.998	0.99 (0.41–2.85)
**Fat Graft Use**	0.063	1.86 (0.96–3.57)	0.233	1.55 (0.75–3.15)
**Fascia Lata Use**	0.152	1.61 (0.83–3.09)	0.347	1.39 (0.69–2.74)
**Lumbar Drain Use**	0.118	1.68 (0.88–3.23)	0.287	1.45 (0.73–2.86)

The predictors were entered as binary categorical variables where the reference group represents the absence of the factor, specifically Nasoseptal Flap Use (Used vs. Not Used), Fat Graft Use (Used vs. Not Used), Fascia Lata Use ( Used vs. Not Used), Lumbar Drain Use (Used vs. Not Used). OR, Odds Ratio; CI, Confidence Interval;

* *p* value < 0.05 was considered significant.

**Table 5:** Surgeon-Reported Factors for CSF Leak Risk and Management Practices.

Category	Factor	Average Rank	Importance/Preference
**Pre-operativeRisk Factors**	Prior surgery	1.80	High Risk
	Patient comorbidities	1.94	High Risk
	Tumor location	2.00	High Risk
	Prior radiation therapy	2.05	High Risk
	Tumor size	2.89	Moderate Risk
	Tumor consistency	4.37	Low Risk
	Vascular encasement	4.52	Low Risk
**Intra-operative Risk Factors**	Intraoperative CSF leak	1.74	High Risk
	Use of lumbar drain	2.95	Moderate Risk
	Extent of resection	3.47	Moderate Risk
	Pituitary stalk preservation	4.52	Low Risk
**Primary Reconstruction Materials**	Nasoseptal flap	1.16	High Preference
	Fascia lata	3.42	Moderate Preference
	Fat graft	3.73	Moderate Preference
	Middle turbinate graft	4.47	Low Preference
	Cartilage graft	4.89	Low Preference
Factors Influencing Reconstruction			
Factor	Score		Importance / Preference
Surgeon preference	12/19		High Influence
Tumor location	11/19		High Influence
Esposito graded repair approach to CSF leaks	11/19		High Influence
Prior treatment	11/19		High Influence
Tumor size	11/19		High Influence
**Management of Confirmed Postoperative Leak**			
**Intervention**	**Average Order**		**Order of Intervention**
Imaging confirmation	2.17		1
Endoscopic visualization	2.29		2
Antibiotic coverage	3.39		3
Lumbar drain trial	3.41		4
Immediate return to surgery	3.56		5

CSF = Cerebrospinal Fluid; Rank order: High Risk/Preference = 1-2.50, Moderate Risk/Preference = 2.514.00, Low Risk/Preference = 4.01=5.00
